# Fabrication of Poly(Lactic Acid)@TiO_2_ Electrospun Membrane Decorated with Metal–Organic Frameworks for Efficient Air Filtration and Bacteriostasis

**DOI:** 10.3390/polym16070889

**Published:** 2024-03-24

**Authors:** Minggang Lin, Jinlin Shen, Qiaonan Qian, Tan Li, Chuyang Zhang, Huan Qi

**Affiliations:** 1 Institute of Smart & Ecological Textile, Quanzhou Normal University, Quanzhou 362002, China; 107552104255@stu.xju.edu.cn (M.L.); chuyangzhang@qztc.edu.cn (C.Z.); 2 College of Textile and Apparel, Xinjiang University, Urumqi 830046, China; 3 College of Textile and Apparel, Quanzhou Normal University, Quanzhou 362002, China

**Keywords:** electrospinning, poly(lactic acid), ZIF-8, bimodal diameter distribution, air filtration

## Abstract

The development of high-performance filtration materials is essential for the effective removal of airborne particles, and metal–organic frameworks (MOFs) anchored to organic polymer matrices are considered to be one of the most promising porous adsorbents for air pollutants. Nowadays, most air filters are generally based on synthetic fiber polymers derived from petroleum residues and have limited functionality, so the use of MOFs in combination with nanofiber air filters has received a lot of attention. Here, a conjugated electrostatic spinning method is demonstrated for the one-step preparation of poly(lactic acid) (PLA) nanofibrous membranes with a bimodal diameter distribution and the anchoring of Zeolitic Imidazolate Framework-8 (ZIF-8) by the introduction of TiO_2_ and in situ generation to construct favorable multiscale fibers and rough structures. The prepared PLA/TZ maintained a good PM2.5 capture efficiency of 99.97%, a filtration efficiency of 96.43% for PM0.3, and a pressure drop of 96.0 Pa, with the highest quality factor being 0.08449 Pa^−1^. Additionally, ZIF-8 was uniformly generated on the surface of PLA and TiO_2_ nanofibers, obtaining a roughened structure and a larger specific surface area. An enhanced filtration retention effect and electrostatic interactions, as well as active free radicals, can be generated for the deep inactivation of bacteria. Compared with the unmodified membrane, PLA/TZ prepared antibacterial characteristics induced by photocatalysis and Zn^2+^ release, with excellent bactericidal effects against *S. aureus* and *E. coli*. Overall, this work may provide a promising approach for the development of efficient biomass-based filtration materials with antimicrobial properties.

## 1. Introduction

With the development of the economy and the deepening of urbanization, industrial emissions have caused air pollution. In recent years, people’s awareness of environmental protection and health has gradually increased, bringing air pollution to the center of society’s attention [1]. Particulate matter (PM) is the primary component of air pollution. PM0.3 and PM2.5 are defined as particulate matter with an aerodynamic diameter less than or equal to 0.3 or 2.5 μm, respectively. The higher the concentration in the air, the more polluted the air [2]. Due to its small particle size, they can remain suspended in the air for an extended period, forming aerosols that can easily enter the respiratory tract and are more harmful than larger particles [3]. Microorganisms, including viruses and bacteria, can attach to particulate matter, potentially spreading disease [4,5]. Wearing masks to filter droplets can help intercept viruses and serve as a crucial tool in preventing and controlling outbreaks. Therefore, there is an urgent need for energy-efficient particle cleaning technology to control PM pollution, which is crucial for human health and energy conservation [6,7]. The membrane is the central component of an air filter. Many researchers have developed filtration materials that are highly filtration efficient [8,9], reduce pressure drop [10,11], and enhance functionality [12,13]. Therefore, the design and development of high-quality filtration membranes are critical for the prevention and control of PM2.5. Fiber filtration membranes are currently a research hotspot in air filtration materials due to their high filtration efficiency and good air permeability.

The main technologies used to produce nanofibers are meltblown and electrostatic spinning [14,15]. Filter membranes are typically made from meltblown polypropylene nonwovens with micron-sized fiber diameters. These membranes typically require corona electret treatment, which has become the predominant technology for producing commercial masks [16,17]. However, the main drawback is the dissipation of charge during daily use, which results in the unstable filtration of the masks. The nanofiber filtration membrane prepared using the electrospinning method possesses characteristics such as a small fiber diameter [18], a large specific surface area [19], and high porosity [20], making it highly suitable for effectively filtering PM2.5 particles [21]. At the same time, traditional polymer materials used for filtration media are typically derived from non-renewable oil sources. Once used, these materials can only be incinerated or disposed of in landfills, creating an environmental burden [22]. As a result, there is a growing concern for the development and production of environmentally friendly nonwoven filtration materials [23,24]. PLA is a common biodegradable material that decomposes into carbon dioxide and water through the action of microorganisms in natural environments. Therefore, PLA products are also widely used in the fields of water separation [25], air filtration, and healthcare [26,27].

Currently, protective fibrous materials function mainly as a physical shield [28]. However, when infected with harmful microbes during and after use, such as viruses, these materials are vulnerable to secondary contamination and transmission [29,30]. As the most often used personal protective equipment, respirators with antibacterial qualities are essential in various situations [31,32]. Two widely utilized techniques to add active protective functions to protective materials are the incorporation of antimicrobial agents into fiber protective materials or the application of antimicrobial agents to their surfaces [33,34,35]. The resulting antimicrobial fiber protective materials can be made using either approach, but they still have significant drawbacks when it comes to obtaining antimicrobial and antiviral effects. The coating that results from applying antimicrobial chemicals to protective fibers has poor durability and weak adherence [36,37].

Of all the photocatalysts, TiO_2_ is the most stable and cost-effective. It has photocatalytic properties that allow it to decompose adsorbed hazardous substances into harmless ones under light irradiation, generating free radicals capable of destroying many microbial bacteria [38,39]. MOFs are a class of nitrogen- or oxygen-containing polydentate organic ligands and metal centers that self-assemble through coordination, forming a highly ordered crystal structure [40,41]. They are extensively utilized in pollutant adsorption, catalysis, and antibacterial applications. Among them, ZIF-8 is a typical MOF known for its permanent pores, high porosity, large specific surface area, and excellent hydrothermal stability [42,43]. Significantly, the unbalanced metal ions and defects in ZIF-8 can polarize the PM surface, enhancing the electrostatic adsorption of PM and exhibiting good antibacterial activity [44]. In light of the aforementioned properties, ZIF-8 has emerged as a prominent material in the field of air filtration [45,46]. Antimicrobial properties are an important issue for current air filters, and although some researchers have developed nanofiber membranes with antimicrobial properties using electrospinning, cumbersome processes or expensive equipment still hinder their wide application [47].

In this study, we present a facile strategy for synthesizing filter membranes with bimodal diameter distributions that exhibit excellent biodegradability and antimicrobial properties. The membrane is composed of biodegradable polylactic acid and is prepared using conjugated electrostatic spinning to generate two different fiber diameters for random mixing in filters, which improves filtration performance and significantly reduces resistance. TiO_2_ and ZIF-8 nanoparticles are added to the fibers to enhance their surface roughness and provide synergistic antimicrobial properties. To confirm the integration advantages of TiO_2_ and ZIF-8 nanoparticles, the filtration performance and antimicrobial properties of electrospun fibers with and without MOF modification were investigated and evaluated in detail. It was demonstrated that the nanoparticles could be uniformly encapsulated on the electrospun fibers, and the focus was on the filtration performance, air permeability, and durability. The production of biodegradable and antimicrobial PLA filters has great potential for applications in healthcare and high-efficiency air filtration.

## 2. Materials and Methods

### 2.1. Materials

Poly(lactic acid) (PLA, REVODE110) was purchased from Zhejiang Haizheng (Taizhou, China) Co., Ltd. Ethyl acetate (EA) and N, N-dimethylformamide (DMF) were purchased from Xilong Scientific (Shantou, China) Co., Ltd. TiO_2_ (20 nm) was purchased from Beasley (Suzhou, China) Co., Ltd. Zinc acetate dihydrate (Zn(CH_3_COO)_2_·2H_2_O) and 2-methylimidazole (2-MI) were purchased from Aladdin Biochemical Co., Ltd., Shanghai, China. Deionized water was taken from the internal water supply system of the laboratory. All chemicals were analytically pure and used without further purification.

### 2.2. Preparation of PLA and PLA/TZ Membranes

**Figure 1:**
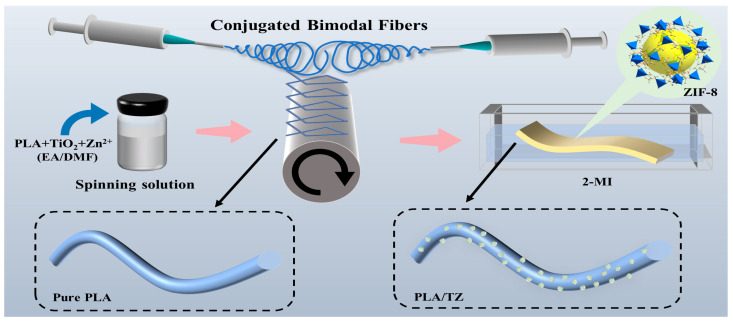
Schematic illustration of fabrication process of PLA/TZ.

The preparation procedure of PLA/TZ membranes is shown in Figure 1. PLA particles were dissolved in a 7:3 (*v*/*v*) solvent mixture of EA and DMF to prepare a 12.0 wt.% solution of PLA. Subsequently, TiO_2_ was added to the solution at concentrations of 5 wt.% of the PLA mass. The mixture was stirred continuously at 75 °C for 24 h. After adding Zn(CH_3_COO)_2_·2H_2_O to the above solution and stirring for 2 h, the mass ratio of PLA to Zn(CH_3_COO)_2_·2H_2_O was 1:1. Electrospinning was performed using an electrostatic spinning device (SS-X3, Yongkang Co. Ltd., Beijing, China). Conjugate electrospinning was performed using two 21-gauge flat-tip needles placed symmetrically and controlled by positive and negative voltages [48]. The solution was fed at 1.0 mL/h and 3.0 mL/h, respectively. The distance from the needles to the receiving rollers was 10.0 cm. The applied electric field ranged from 10.0 kV to 15.0 kV. The ejected fibers were stretched by an electric field and then deposited onto a metal collector roller, which was wrapped with a laboratory-prepared PLA spunbonded nonwoven fabric. A three-dimensional, fluffy, conjugated bimodal diameter-distributed membrane was produced in the experiment. The metal collector was rotated at a speed of 300 rpm and positioned a little bit below the needle. The membrane was then dried at 50 °C for 24 h to remove any residual solvent, named PLA/T.

The 2-MI was dissolved in deionized water and ultrasonically agitated for 30 min. The prepared PLA/T membranes were then immersed in the solution and reacted at room temperature for 4 h. During the process, Zn^2+^ crystal species on the surface of PLA fibers coordinated with 2-MI in the solution. Subsequently, they were removed, thoroughly rinsed, and dried in an oven at 40 °C to obtain the final PLA/TZ membranes.

### 2.3. Characterization

The surface morphology of the bimodal filter was investigated using a scanning electron microscope (TESAN MIRA LMS, Tescan China Ltd., Shanghai, China), sputter coating the samples with gold. The diameter and distribution of the nanofibers were analyzed using Nano Measure 1.2 software. The surface chemical elements were analyzed using an energy dispersive X-ray (EDX) detector (Thermo Scientific Helios 5 CX, Thermo Fisher Scientific, Waltham, MA, USA). The Fourier transform infrared (FT-IR, Thermo Scientific Niolet iN10, Thermo Fisher Scientific, USA), ranging from 600 to 4000 cm^−1^, of the samples was recorded before and after modification, and changes in the spectra were observed. The crystal structure of the samples was analyzed using X-ray diffraction (XRD, Rigaku Analytical Devices Inc., Tokyo, Japan), with scattering angles ranging from 5 to 90°. Analyzing the elemental and electronic states of the sample surfaces was performed using X-ray photoelectron spectroscopy (XPS, Thermo Fisher Scientific, America) with an excitation source of Al Kα-1486.6 eV. The narrow-spectrum scan has a pass energy of 50 eV and a step size of 0.1 eV. The air permeabilities of the filter samples were evaluated on an air permeability tester (YG461E, Quanzhou Meibang Instrument Co., Ltd., Quanzhou, China), in accordance with the ASTM D 737 standard [49].

### 2.4. Filter Performance Evaluation

The particle filtration efficiency tester (DR251XL, Wenzhou Darong Textile Instrument Co., Ltd., Wenzhou, China) was used to measure the filtration performance and pressure drop. The filtration test equipment contained the test membrane with an effective area of 100 cm^2^. An air compressor generated the carrier airflow, which was continuously adjustable within the range of 10–90 L/min. The aerosol used was an electrically neutral NaCl solution with a concentration of 2.0 wt.%, produced through a dust generator and dried to pass through the test sample. The NaCl aerosol had a normal particle size distribution, with a median diameter of 75 nm and a geometric standard deviation of less than 1.86. To calculate filtration efficiency, the concentration of aerosol is measured both upstream and downstream using the built-in spectrophotometer. The filtration efficiency (η) can be calculated using the following equation:

(1) $$\eta =\frac{C_{\mathrm{up}}-C_{\mathrm{down}}}{C_{\mathrm{up}}}\;\times \;100\%$$

where C_up_ and C_down_ are the concentrations of the NaCl aerosols in the upstream and downstream flow, respectively.

Pressure drop is determined by measuring the differential gas pressure between the upstream air inlet and the downstream air outlet of the filtration material. In order to better evaluate the overall filtration performance of the filter media, the quality factor (QF) is used to evaluate when a higher QF indicates that the material has a better filtration performance. The QF can be calculated as the following equation:

(2) $$\mathrm{QF}=-\frac{\mathrm{Ln}\left(1-\eta \right)}{\Delta P}$$

where ΔP represents pressure drop.

The filtration loading test was conducted to examine the dynamic filtration properties in accordance with GB 2626-2019 [50]. The concentration of NaCl aerosol was adjusted to a maximum of 20.0 mg/m^3^, and the airflow rate was set to a maximum of 85.0 L/min. When the pressure drop reaches a certain value, the increase in weight of the air filter reflects its dust holding capacity. The dust holding capacity was measured by a filter loading test that stops when the initial pressure drop doubles during the loading test, and it was calculated by weighing the mass of the deposited particles per unit area of the filter. Meanwhile, in order to evaluate the performance of the filter at effectively capturing PM, an independent filter tester was designed and constructed to simulate real-life haze conditions. The test membrane was placed in the middle of two transparent boxes with continuous ventilation, and a cigarette was lit on one side of the membrane. During the combustion process, the concentration of smoke was observed on both sides of the membrane. The PM concentration was measured using a dust particle counter (Airhug-CP-15, Beijing Yishan Technology Co., Ltd., Beijing, China) to determine the actual filtration of the PM concentrations on both sides of a burning cigarette.

### 2.5. Antibacterial Performance Evaluation

Antibacterial tests were performed according to the standard GB 15979-2002, and the tested strains were Escherichia coli (*E. coli*, ATCC 25922, Gram-negative) and Staphylococcus aureus (*S. aureus*, ATCC 25923, Gram-positive). The specific experimental procedure was in accordance with reference [51,52].

## 3. Results and Discussion

### 3.1. Morphological Observation of Membranes

**Figure 2:**
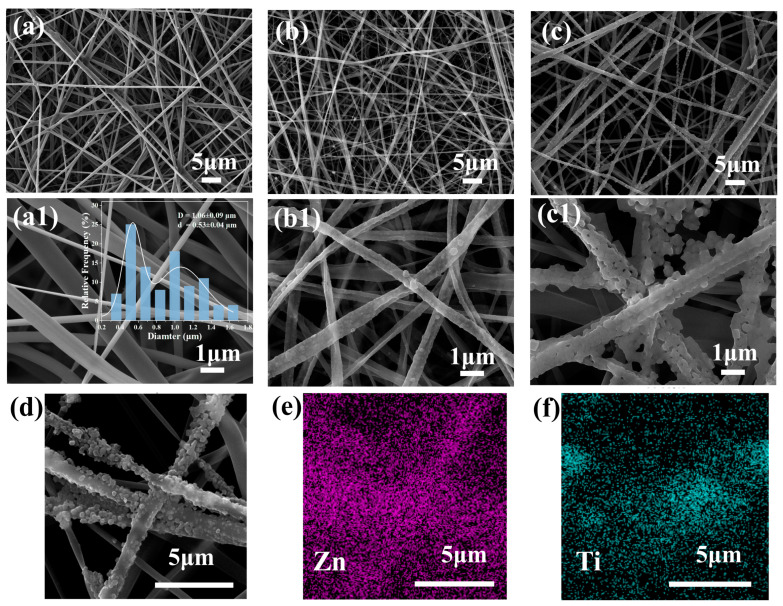
The SEM images of PLA (**a**,**a1**), PLA/T (**b**,**b1**), and PLA/TZ (**c**,**c1**) and (**d**) EDS mapping of PLA/TZ showing the distribution and atomic proportions of (**e**) Zn and (**f**) Ti elements.

SEM was used to study the morphology of the as-developed electrospun nanofiber membranes. Figure 2a–c display the SEM images of pure PLA, PLA/T, and PLA/TZ nanofiber membranes. The distributions of the bimodal diameters of the fibers of pure PLA were 1.06 ± 0.09 μm and 0.53 ± 0.04 μm, respectively. The fibers on the surface of the PLA membranes were uniform, smooth, and randomly oriented. The addition of TiO_2_ resulted in a finer and more uniform distribution of the fiber diameters. This was attributed to the dilution of the PLA concentration by TiO_2_ and the increased electrical conductivity of the electrospinning precursor solution [53]. Although the bimodal distribution of the fiber diameters was somewhat weakened, a homogeneous diameter with a still bimodal distribution can be clearly seen in Figure 2b,c. The weakening of the bimodal distribution is mainly due to viscosity, which causes the uneven splitting of the polymer liquid during the electrospinning process. The surface of the PLA nanofibers obtained by electrospinning is smooth and uniform. The addition of TiO_2_ significantly improves the roughness of the fiber surface. The surface of the PLA/TZ nanofiber membrane is covered with a layer of dense ZIF-8 particles. The protrusion of the nanoparticles increases the surface roughness of the fiber membrane, thereby enhancing the effective surface area of the membrane. In addition, hydrogen bonding between polylactic acid (PLA) and 2-methylimidazole promotes the uniform distribution of ZIF-8 on the fiber surface. Small-sized ZIF-8 nanoparticles were uniformly loaded and wrapped onto the fiber surface, thereby further enhancing the performance of the fiber membrane. The composite membrane was enhanced with high photocatalytic activity and improved barrier performance by dispersing TiO_2_ and ZIF-8 on the fiber surface.

The EDS spectrogram confirmed the uniform appearance of TiO_2_ and ZIF-8 on the PLA fibers without agglomeration, further indicating their presence on the fiber surface. Environmental factors such as humidity and temperature may cause variations in electrospun fibers in addition to those caused by the nature of the solution and processing conditions.

### 3.2. Characterization of Membranes

**Figure 3:**
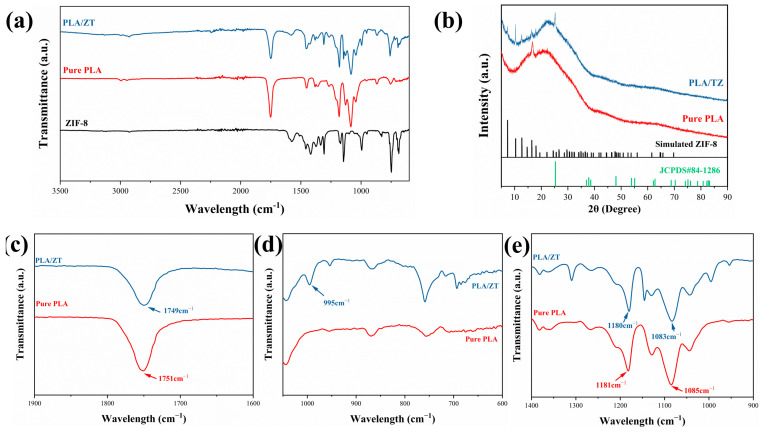
(**a**) The FTIR spectra of PLA, PLA/T, and PLA/TZ; (**b**) the XRD patterns of PLA and PLA/TZ and the FTIR spectra in the range of (**c**) 1900−1600 cm^−1^, (**d**) 1000−600 cm^−1^, and (**e**) 1400−900 cm^−1^.

The surface chemical structure of the nanofiber membranes was characterized using FTIR. The full-range FTIR spectra in the 4000–650 cm^−1^ region are shown in Figure 3a, while the local spectra are shown in Figure 3c–e. The telescopic vibrational absorption peaks of C=O in the PLA chains were located at 1751 cm^−1^. The absorption peaks of C-H and C-H were located at 1454 cm^−1^. The symmetric and asymmetric telescopic vibration peaks of C-O-C were located at 1181 cm^−1^ and 1085 cm^−1^, respectively, and they gradually shifted to 1180 and 1185 cm^−1^ (Figure 3c,e). The telescopic vibration absorption peak of C=O shifted to 1749 cm^−1^, mainly due to the introduction of TiO_2_. The addition of ZIF-8 facilitated the interaction between numerous carbonyl groups on the PLA chain and the oxygen atoms located at the edge and surface of TiO_2_. Meanwhile, the nanofibrous membranes loaded with ZIF-8 exhibited peaks corresponding to aromatic and aliphatic C-H stretching vibrations at 2900 cm^−1^ and 3100 cm^−1^, respectively. In addition, a peak of C=N stretching vibration was observed at 1570 cm^−1^. For PLA/TZ, characteristic bending vibration peaks attributed to the imidazole ring (due to the introduction of ZIF-8) were observed at 758 cm^−1^. The successful incorporation of TiO_2_ and ZIF-8 nanostructures into PLA nanofibers was confirmed by the migration and intensity changes in the characteristic peaks, as well as the emergence of new characteristic peaks.

The crystalline structure of the nanofiber membrane was analyzed using XRD. The pure PLA nanofiber membrane exhibited a sharp peak at 17.6°, attributed to the diffraction of the (200/110) crystal plane, indicating the characteristic presence of orthorhombic crystals in the pure PLA nanofibers. The ZIF-8 particles exhibited characteristic peaks at 7.2°, 10.4°, 12.6°, and 18.0°, corresponding to the (011), (002), (112), and (222) crystal plane diffractions, respectively. At 25.3°, titanium dioxide exhibited a distinct characteristic peak corresponding to the (101) crystal plane diffraction [54]. The simultaneous appearance of characteristic diffraction peaks of PLA, TiO_2_, and ZIF-8 in the spectrogram of PLA/TZ fiber membranes further demonstrates the successful growth of TiO_2_ and ZIF-8 on the surface of the PLA fibers.

**Figure 4:**
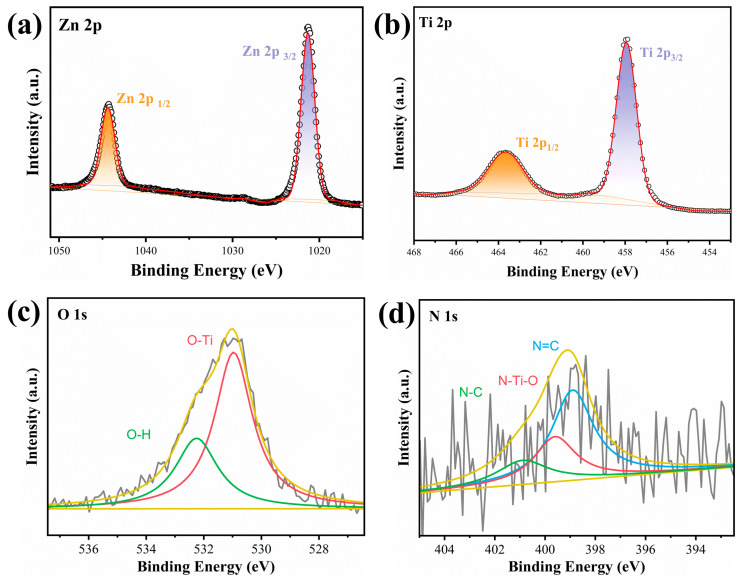
Fitting spectrum of the XPS: (**a**) Zn 2p, (**b**) Ti 2p, (**c**) O 1s, and (**d**) N 1s.

The surface chemical bonding states of PLA/TZ were investigated using XPS. The high-resolution spectra indicate that the membrane acquired Zn, Ti, O, and N elements, as shown in Figure 4. The Zn 2p orbital spectrum in Figure 4a shows peaks at 1044.7 eV and 1021.6 eV, corresponding to Zn 2p3/2 and Zn 2p1/2 peaks, respectively, indicating the presence of Zn^2+^. The Ti 2p XPS spectrum displays two peaks (Figure 4b) at binding energies of 464.4 eV and 458.7 eV, corresponding to Ti 2p3/2 and 2p1/2, respectively. The spin-orbit splitting energies of these two peaks are about 5.7 eV, indicating that Ti^4+^ is in a normal state in the PLA/TZ. This suggests the presence of oxidized Ti^4+^ in the membrane, which is similar to that of TiO_2_. Figure 4c O 1s spectrum displays two peaks at 530.1 eV and 532.3 eV, which can be attributed to the lattice oxygen (Ti-O bond) and external -OH groups or H_2_O molecules absorbed on the sample surface [55]. The high-resolution mapping of N 1S in Figure 4d shows three N 1s peaks at 398.9 eV, 399.6 eV, and 400.9 eV, which were processed by peak splitting and consisted of N=C, N-Ti-O, and N-C bonds of the imidazole ring of ZIF-8 [56,57]. The presence of N-Ti-O bonds suggests that some of the oxygen atoms on the surface of TiO_2_ were replaced by nitrogen atoms of 2-MI. FTIR, XRD, and XPS analyses demonstrate the successful in situ growth and ordered self-assembly of ZIF-8 into macroscopically robust fibrous membranes along the PLA fiber backbone. These findings provide additional evidence of the successful production of PLA/TZ nanofiber membranes.

### 3.3. Filtration Properties

**Figure 5:**
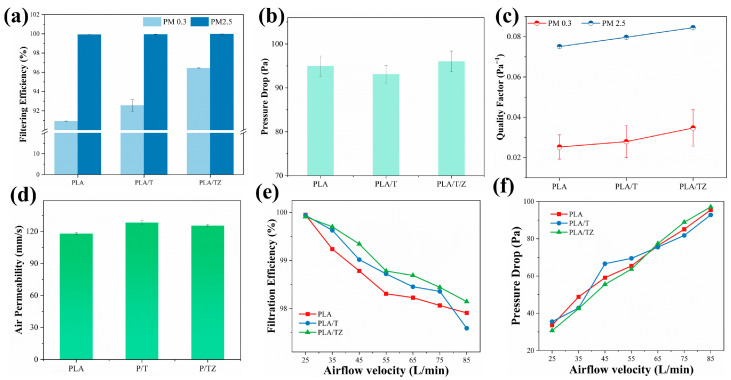
(**a**) The filtration efficiency for PM0.3 and PM2.5, (**b**) pressure drop, (**c**) quality factor, (**d**) air permeability of PLA, PLA/T, and PLA/TZ, changes in (**e**) filtration efficiency, and (**f**) pressure drop under different air flow rates of PLA, PLA/T, and PLA/TZ.

The concentration of sodium chloride aerogel particles was measured upstream of the generator and downstream of the receiver sensor in order to assess the filtration performance and efficiency. This study extensively investigated the filtration efficiency at a flow rate of 85 L/min for PM2.5 and PM0.3, using filtration efficiency, pressure drop, and the QF as evaluation parameters. Figure 5a illustrates the impact of air filtration performance with varying loadings. The filtration efficiency of pure PLA was 90.92% for PM0.3, while PLA/T and PLA/TZ achieved 92.56% and 96.43%, respectively. The filtration efficiencies for PM2.5 were 99.92%, 99.94%, and 99.97%, respectively. The fiber membranes loaded with TiO_2_ and ZIF-8 both effectively improved the filtration efficiencies, with a more significant effect observed in PM2.5. The introduction of titanium dioxide is attributed to the increase in solution conductivity, which results in a reduction in the fiber diameter. This reduction can effectively increase the physical interception of dust particles. It is interesting to note that the simultaneous introduction of ZIF-8 and TiO_2_ effectively raised the fibers’ roughness, which raised the filtration efficiency even further. Previous investigations came to a similar conclusion about this phenomenon [58]. Figure 5b shows that the corresponding pressure drops of PLA, PLA/T, and PLA/TZ were 95.0 Pa, 93.1 Pa, and 96.0 Pa, respectively. The nanoparticles on the surface did not cause a significant change in the pressure drop. When considering filter materials, it is important to strike a balance between filtration efficiency and pressure drop. The QF represents the overall performance of filtration and is related to both filtration efficiency and pressure drop. Figure 5c displays the QFs for PM0.3 and PM2.5, which were 0.02526 Pa^−1^, 0.02790 Pa^−1^, and 0.03470 Pa^−1^ and 0.07509 Pa^−1^, 0.07968 Pa^−1^, and 0.08447 Pa^−1^, respectively. The bimodal diameter distribution of the constructed and loaded nanoparticles in the PLA membrane resulted in a high QF of up to 0. 08447 Pa^−1^ thanks to the effective interception of nanofibers and micron fibers by conjugated electrostatic spinning to form 3D structures with fluffiness. The roughness was improved, and the slip effect was enhanced by the addition of ZIF-8, which was further intensified by its incorporation. This phenomenon can be verified by the air permeability test. Figure 5d shows that PLA/TZ has the highest average air permeability at 125.59 mm/s, while pure PLA has the lowest at 118.09 mm/s.

The effect of different airflow rates on the filtration efficiency for PM2.5 removal is shown in Figure 5e,f. As the airflow rate rises from 25.0 L/min to 85.0 L/min, the filtering efficiency progressively declines; the PLA/TZ falls from 99.92% to 98.15% while staying above 95.0%. In addition, the pressure drop increased from 42.5 Pa to 97.1 Pa, in accordance with Darcy’s law [59]. The correlation between pressure drops and air velocity suggests that the membrane material has open air channels and good air permeability. Both the introduction of TiO_2_ and the loading of ZIF-8 follow a consistent pattern of change. Mechanical interception effects such as interception, inertial collision, Brownian motion, and gravity deposition are primarily observed in air filtration. Interception and gravity effects play a more prominent role in capturing larger particles due to their size and weight. The PLA/TZ membrane is most effective at filtering particles of 2.5 μm, making it suitable for everyday use.

### 3.4. Loading Performance

**Figure 6:**
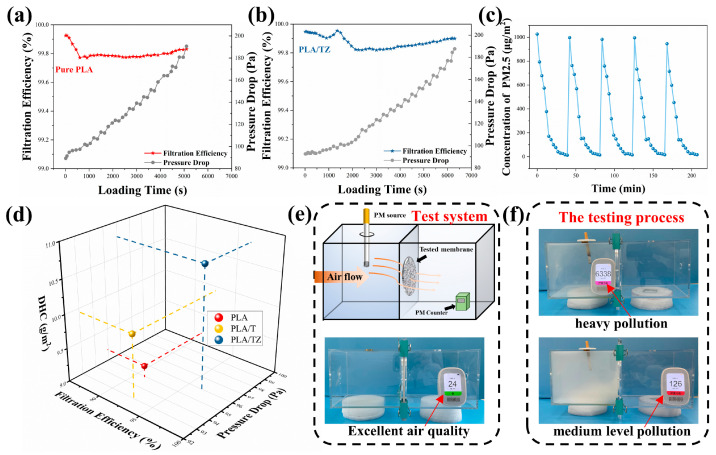
The filtration efficiency and pressure drop during the loading test of (**a**) PLA/TZ and (**b**) PLA, (**c**) 5 cycles of circulating filtration PM2.5, (**d**) dust holding capacity of filtration performance, (**e**) picture of PM2.5 filter device and filtration test system with the burning cigarette, and (**f**) PM2.5 measurements that simulate the actual smoke testing process.

In a practical environment, it may be necessary for the filter media to provide continuous filtration for a long time. Therefore, the stability of the dynamic filtration performance is critical for filter materials. In order to evaluate the filtration stability and durability of membranes, load tests were carried out at a flow rate of 85.0 L/min. The results are shown in Figure 6a,b. During the loading test, the dust collector continuously produced sodium chloride aerosols at a concentration of 20 mg/cm^3^, which passed through the test membrane and were intercepted and deposited in the filter media. The trapped particles also acted as a deposition site for subsequent new particulate matter, thereby enhancing mechanical interception. During the loading test period, the filtration efficiency of PLA/TZ initially decreased and then increased over time, while the filtration resistance gradually increased. It is evident that PLA/TZ has higher filtration stability than PLA throughout the entire loading process, and it provides better overall filtration performance for prolonged use. When the resistance has doubled from the initial level, the loading test is stopped. The weight change in the membrane is then measured to determine the dust holding capacity. This is an important indicator of filtration performance and is directly related to the replacement cycle of the filter material. The dust holding capacity significantly influences the service life of the air filter as it directly affects the frequency of filter replacements. Figure 6d shows a comparison of the dust holding capacity of PLA, PLA/T, and PLA/TZ. The dust holding capacity of PLA, PLA/T, and PLA/TZ is 9.17 g/m^2^, 9.93 g/m^2^, and 10.79 g/m^2^, respectively. This indicates that PLA/TZ has a higher dust holding capacity than the other two, and a higher filtration efficiency based on comprehensive performance evaluation. The dust holding capacity of PLA/TZ reflects its retention capacity and service life. The bimodal distribution and coarser fibers of the construction facilitate airflow, providing longer channels and more deposition sites for small particles to be trapped. The rough structures can increase the number of settlement sites. As a result, the filter media can effectively trap more dust particles, and if it takes longer to reach a given and comparable level of pressure drop, the filter may have a longer service life.

Smoke removal efficiency is a crucial factor to consider when evaluating filtration materials. It is particularly important to assess their ability to purify haze larger than PM2.5. To determine the purification efficiency of PLA/TZ in removing smoke, we simulated real-world conditions using a homemade device (refer to Figure 6e). The left side of the box was filled with the generated PM, while the PM2.5 particle counters on both sides of the membrane detected them separately, as shown in Figure 6f. The PLA/TZ effectively intercepted and absorbed the PM, resulting in a clear and transparent right side of the box. This indicates that the prepared membrane has good filtration performance, which is maintained even after five cycles in Figure 6c. This confirms that PLA/TZ has superior PM2.5 removal capability and purification efficiency, indicating a strong potential for use in masks.

### 3.5. Antibacterial Performance

**Figure 7:**
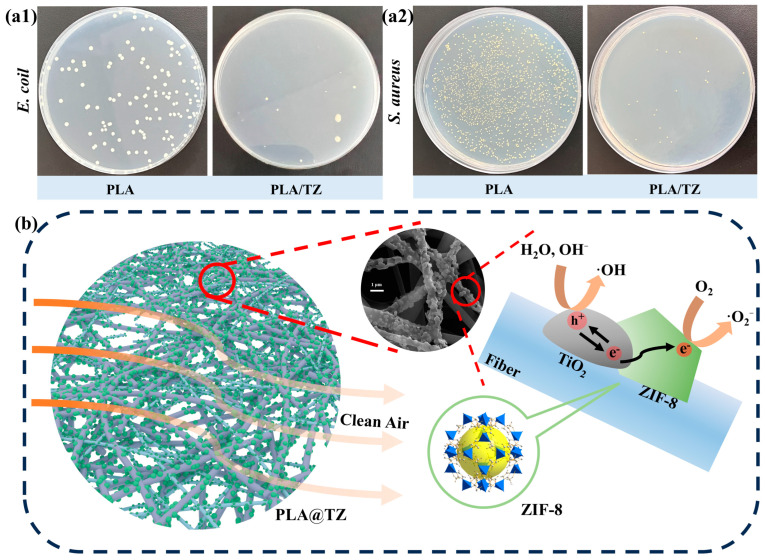
(**a1**,**a2**) The antibacterial properties of PLA and PLA/TZ against E. coli and *S. aureus*, (**b**) and the schematic diagram of PM filtration by PLA/TZ and possible photocatalytic antimicrobial pathways.

Antimicrobial activity is a critical factor in the evaluation of protective fiber materials for active protection. In previous reports, TIO_2_ and ZIF-8 have also demonstrated antimicrobial properties for medical protection applications. In this study, antimicrobial PLA-based fibers doped with TiO_2_ and loaded with ZIF-8 were prepared and tested for their antimicrobial activity against Gram-negative (*E. coli*) and Gram-positive (*S. aureus*) bacteria. The results showed that PLA/TZ exhibited significantly higher antimicrobial properties than PLA, as shown in Figure 7(a1,a2). The bactericidal rate was calculated by the count method, and the inhibitory effect on *E. coli* and *S. aureus* was 98.3% and 97.6%, respectively. The demonstrated bactericidal activity predicts the great application prospects of MOF protection for ring-breaking and bacterial killing. Figure 7b illustrates the schematic diagram of the synergistic PM filtration mechanism of TiO_2_ and ZIF-8. ZIF-8 crystals have a high specific surface area and abundant adsorption sites, enabling them to adsorb fine particles on the PLA/TZ membrane as they pass through the visible-light-irradiated membrane. As a result, pollutants are trapped in the fibers. Under light conditions, both TiO_2_ and ZIF-8 can be activated, and the ·O^2−^ and ·OH generated by PLA/TZ can lead to the oxidative decomposition of PM2.5 [60]. In addition, the heterostructure formed by ZIF-8 and TiO_2_ promotes charge separation and reduces the bandgap. The charge carriers are induced by light, and the filter membrane generates ROS. ROS are free radicals with strong reducing and oxidizing capabilities, which can damage the phospholipid layer of the cell’s outer membrane and react with many organic substances inside the cell [61,62].

## 4. Conclusions

In conclusion, we present a strategy for the preparation of bimodal diameter-distributed polylactic acid filtration membranes by introducing TiO_2_ and the in situ anchoring of ZIF-8 coatings. This approach is straightforward and simple to use; it may successfully increase the roughness by uniformly embedding nanoparticles into electrospun fibers. In particular, the filtration results confirm that the developed ZIF-8 embedded nanofiber filter can effectively capture submicron particles. The pressure drop remained stable, while the filtration efficiency was improved, ranging from 90.92% to 96.43% for PM0.3, especially for PM2.5, with good interception performance, obtaining the highest quality factor of 0.08449 Pa^−1^. Furthermore, the stability resistance test demonstrated an extended service life in real-world application conditions, and it exhibited some degree of antibacterial performance. In general, this research offers significant perspectives on the creation of materials for nanofiber air filtration.

## Data Availability

The data presented in this study are available on request from the corresponding author.
